# Renal Riddles Unveiled: Decoding Homogeneous Kidney Enlargement in Multiple Myeloma

**DOI:** 10.7759/cureus.50269

**Published:** 2023-12-10

**Authors:** Ali Zafar Sheikh

**Affiliations:** 1 Radiology, Shaukat Khanum Memorial Cancer Hospital and Research Centre, Lahore, PAK

**Keywords:** chemotherapy, renal cell carcinoma, extramedullary plasmacytoma, immunoglobulin, cytogenetically, renal plasmacytoma, plasma cell neoplasms, multiple myeloma

## Abstract

Renal plasmacytoma is a rare extramedullary manifestation of plasma cell neoplasms (PCN). We present the case of a 62-year-old male with a history of relapsed refractory multiple myeloma (MM) who developed secondary renal plasmacytoma after an eight-year remission period. Radiological findings on plain CT raised concern for the most common renal malignancy, i.e. renal cell carcinoma (RCC), while further imagining evaluation with MRI suggested renal lymphoma, highlighting the diagnostic challenge of renal plasmacytoma on imagining. A renal mass biopsy confirmed a kappa-restricted plasma cell tumor, emphasizing the need for accurate differentiation between renal plasmacytoma and other renal malignancies to guide appropriate treatment strategies. Increased awareness of such cases can lead to timely recognition and tailored management for this rare entity.

## Introduction

Multiple myeloma (MM) was once considered a distinct disease but is now recognized as part of a spectrum of plasma cell neoplasms (PCN) [1,2]. PCN involves uncontrolled proliferation of mature B-cells and plasma cells, leading to various symptoms [1]. According to the 2022 International Consensus Classification (ICC), PCN includes MM, plasmacytoma, abnormal immunoglobulin deposition diseases, and non-immunoglobulin M (IgM) monoclonal gammopathy of undetermined significance (MGUS) [2]. Extramedullary plasmacytoma (EMP) can coexist with MM or occur independently [3]. Though the diagnosis of renal plasmacytoma may be suggested on imaging, confirmation necessitates a comprehensive diagnostic approach [4-7]. MRI diffusion restriction-based sequences aren't very helpful in distinguishing between renal plasmacytoma and renal lymphoma, even though they're usually reliable for lymphoproliferative disorders [8,9]. The similarity of the main findings with common kidney issues makes us unsure about the first diagnosis, highlighting how crucial it is to use imaging to tell renal plasmacytoma apart from other conditions correctly [6,10,11]. Different treatments emphasize the need for accurate identification to personalize patient care: radiotherapy stands as the primary treatment for renal plasmacytoma, radical nephrectomy followed by adjuvant therapy is preferred for renal cell carcinoma, and systemic chemotherapy is key for treating renal lymphoma [12-14].

## Case presentation

A 62-year-old married male presented initially in 2013 with a 10-month history of multiple site bone pains. On further inquiry, there was a history of night sweats, fever, and weight loss. The vital signs were in the normal range and the physical exam was unremarkable. The labs were significant for increased total serum protein, with electrophoresis demonstrating a monoclonal protein band in the gamma region. There was an increased kappa/lambda ratio of 4.21 g/L. The patient was given six cycles of bortezomib, thalidomide, and dexamethasone in 2013 with a complete response toward therapy. However, the patient again presented in 2014 with relapsed refractory multiple myeloma, for which he was given five more cycles of lenalidomide and dexamethasone; however, he was not put on remission maintenance because of neuropathy.

**Figure 1:**
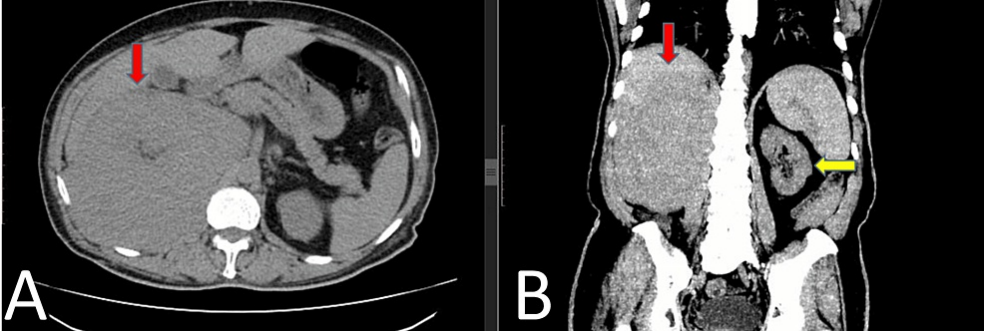
CT scan abdomen and pelvis without contrast Axial (A) and coronal (B) slices demonstrate a solid mass lesion in the retroperitoneal plane completely replacing the right kidney (red arrows). The normal left kidney can also be noted (yellow arrow).

He again presented after a lapse of eight years in February 2022 with a complaint of non-specific abdominal pain, for which he underwent CT abdomen and pelvis without contrast, as the estimated glomerular filtration rate (eGFR) was sub-optimal, with a value of 46.27 mL/min/1.73 m^2^. The study was significant for enlarged right kidney without any significant perinephric fat stranding, and the impression was more in favor of a neoplastic process in particular renal cell carcinoma, rather than infection (Figure 1).

**Figure 2:**
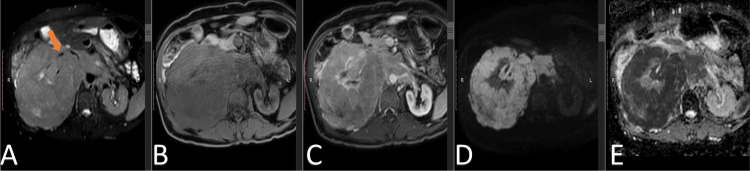
MRI abdomen with contrast Axial sections of the right renal mass at the level of the renal hila demonstrating hyperintense ​T2W fat sat (A), hypointense on mDIXON (B) signals. It shows enhancement on mDixon contrast + (C) and demonstrates restricted diffusion on DWI sb-1000 (D) and dADC (E). It encases the ipsilateral right renal vasculature (orange arrow) without loss of flow void. T2W: T2-weighted; DWI: diffusion-weighted imaging

**Figure 3:**
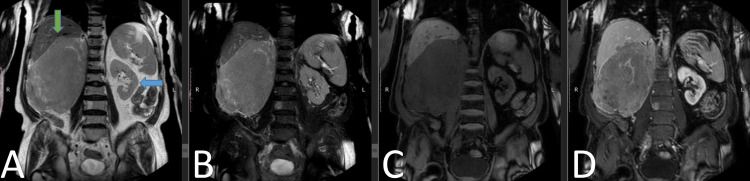
MRI abdomen with contrast Coronal slices demonstrate the right renal mass (green arrow) returning hyperintense signals on T2W (A) and T2W-fat sat (B), hypointense signals on mDIXON (C), and demonstrating contrast enhancement on mDIXON C+ (D). The normal-appearing left kidney may also be seen (blue arrow). T2W: T2-weighted

**Figure 4:**
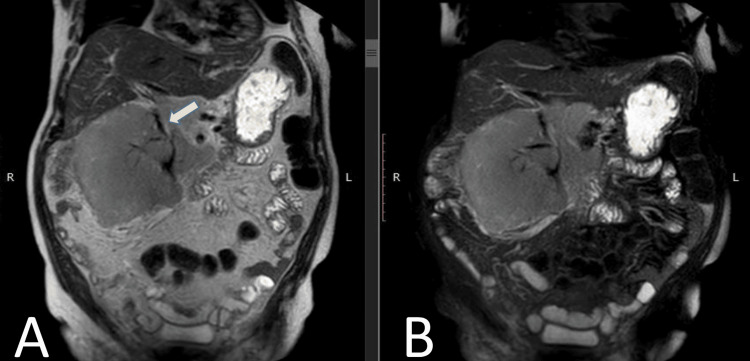
MRI abdomen with contrast Coronal T2W (A) and T2W fat-sat (B) sequences demonstrate the right renal mass encasing the inferior vena cava (white arrow) without any loss of flow voids T2W: T2-weighted

He subsequently underwent an MRI of the abdomen for further characterization, which demonstrated a diffusely enlarged right kidney with a fairly homogeneous signal intensity. The renal mass was hypo-intense on T1WI and intermediate on T2WI, demonstrating homogeneous contrast enhancement on T1W C+ and restricted diffusion on DWI and ADC. The lesion encased the renal vasculature and inferior vena cava without any significant luminal compromise and without any loss of flow-void, a morphological characteristic strongly associated with lymphoma (Figures 2-4).

**Figure 5:**
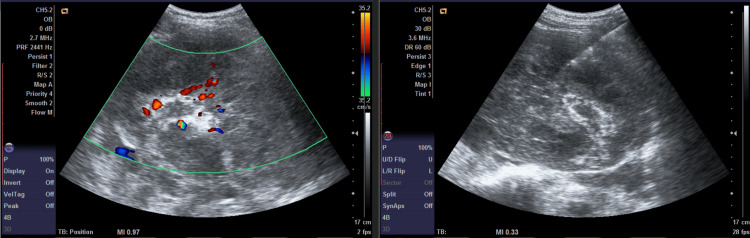
Ultrasound-guided Tru-cut biopsy images of the right renal mass, longitudinal view The right-sided image demonstrates low vascularity of the right renal mass on duplex evaluation while the left-sided image demonstrates the Tru-cut biopsy needle well placed in the mass.

A differential diagnosis of renal lymphoma was suggested. The patient underwent an ultrasound (USG)-guided Tru-cut core biopsy of the mass (Figure 5).

**Figure 6:**
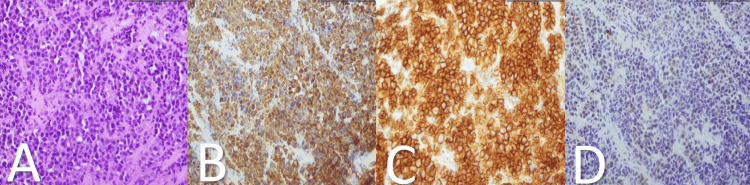
Histopathology images of the renal Tru-cut biopsy Typical plasma cells (A) demonstrate kappa (B) and Cd-138 (C) restriction while no lambda (D) restriction is noted.

The biopsy result indicated a kappa-restricted plasma cell tumor suggestive of renal plasmacytoma (Figure 6).

The patient was initiated on cyclophosphamide, thalidomide, and dexamethasone. After two cycles of chemotherapy, the patient presented in the emergency with febrile neutropenia. He developed disseminated intravascular coagulation with fluid refractory hypotension leading to acute renal injury from which he could not recover.

## Discussion

Multiple myeloma (MM) was once thought of as a distinct disease, but it is now known to be part of a spectrum of disorders that involve cytogenetically different plasma cell neoplasms (PCN) [1,2]. The pathogenesis of PCN is uncontrolled mature, well-differentiated B-cell and plasma cell proliferation, which in itself and by the unrestrained production of immunoglobulin (Ig) causes various signs and symptoms such as osseous lytic lesions, a disruption of normal hematopoiesis, deterioration of renal function, etc. [1].

PCN, according to the 2022 International Consensus Classification (ICC), includes multiple myeloma (MM), plasmacytoma, which further comprises solitary bone plasmacytoma (SBP) and extra-medullary plasmacytoma (EMP), abnormal immunoglobulin deposition, which further comprises light chain amyloidosis and non-amyloid light and/or heavy chain immunoglobulin deposition diseases, and non-IgM monoclonal gammopathy of undetermined significance (MGUS), which further comprises plasma cell type and not otherwise specified (NOS) type [2].

Extramedullary plasmacytoma (EMP) tumors can exist alongside MM, either as the primary characteristic of MM, emerge during the progression of MM, or, in some cases, arise as singular tumors without any other characteristics of MM [3].

EPM occurs when the disease's cells invade other organs or circulate unhindered in the blood after separating from the myeloid tissue [2]. EPM is of two types: most EMPs develop and present as a secondary entity to multiple myeloma or less frequently as a primary entity without any history or relation to MM [4]. Most cases of renal plasmacytoma that have been recorded so far are primary while it is a secondary type in our case report [3]. Imaging alone is unable to differentiate primary renal plasmacytomas from other renal tumors, necessitating the use of blood tests, biochemical analyses, and tissue-based investigations for the complex diagnosis of an EMP [5,6]. According to one study, certain diagnoses require the presence of an extraosseous myelomatous mass and rule out marrow involvement in bone scintigraphy [4].

The usual appearance of renal plasmacytoma is a discrete focal enhancing lesion on both CT scans and MRI [3,7]. The MRI additionally shows restriction on diffusion-weighted images (DWI), though it is of no value in narrowing down the differential as lymphoma and renal cell carcinoma (RCC) also tends to demonstrate diffusion restriction [8,9]. There is also an increasing role for whole-body MRI (WB-MRI) in the PCN, but that too is to quantify the burden of the disease initially and later to look for a response to therapy rather than primarily making the diagnosis [8].

In our case, two notable observations challenged the initial consideration of renal plasmacytoma as the differential diagnosis. First, the occurrence of secondary renal plasmacytoma in patients with multiple myeloma (MM) is significantly rarer compared to RCC or renal lymphoma. Second, the imaging characteristics pointing toward lymphoma, further complicate the differential diagnosis [6,10,11]. The chemotherapy approaches vary between renal lymphoma and renal plasmacytoma. Also, radiotherapy is an alternative for plasmacytoma, whereas chemotherapy is the primary treatment for lymphoma in the kidneys. This divergence in treatment modalities emphasizes the importance of accurately identifying and differentiating between these distinct conditions. Such discernment enables the development of tailored management strategies and ensures optimal care for patients [12-14].

## Conclusions

In summary, this case highlights the rarity and diagnostic challenges of secondary renal plasmacytoma, emphasizing the need for accurate differentiation from renal lymphoma and renal cell carcinoma. Distinct treatment modalities underscore the importance of precise identification. Comprehensive diagnostic approaches and advanced imaging techniques are essential while increased awareness and timely recognition are crucial for optimal patient care and tailored management.
